# Oesophageal squamous cell carcinoma mimicking submucosal tumour

**DOI:** 10.1186/s12876-021-01731-7

**Published:** 2021-04-01

**Authors:** Wen Wang, Dazhou Li, Linfu Zheng, Hongli Zhan

**Affiliations:** 1 Fuzhou General Clinical Medical College of Fujian Medical University, Fuzhou, 350025 China; 2 Department of Gastroenterology, 900Th Hospital of People’s Liberation Army, 156 North Road, West second Ring Road, Gulou District, Fuzhou, 350025 China; 3 Oriental Hospital Affiliated To Xiamen University, Fuzhou, 350025 China

**Keywords:** Oesophageal squamous cell carcinoma, Mimicking, Submucosal tumour

## Abstract

**Background:**

Oesophageal submucosal tumours are usually benign. We report a rare case of esophageal squamous cell carcinoma presenting as a submucosal tumour.

**Case presentation:**

A 58-year-old man undergoing screening oesophago-gastroduodenoscopy was found to have a smooth-surfaced 0.6-cm sized submucosal tumour in the oesophagus 30 cm from the incisor. Endoscopic ultrasonography showed the tumour to be located in the muscularis mucosa; the lesion was heterogeneously hypoechoic and had a clear boundary. With a provisional diagnosis of leiomyoma, the tumour was removed by endoscopic submucosal dissection. Pathological examination showed it to be a moderately differentiated infiltrating squamous cell carcinoma, with normal overlying squamous epithelium. Immunohistochemistry indicated that it was caused by malignant transformation in mucosal glandular duct epithelium. Positron emission tomography–computer tomography showed no tumour spread to any other site. The patient was treated by oesophageal resection.

**Conclusion:**

The clinician should be aware that oesophageal submucosal tumours with smooth overlying mucosa may not always be benign; malignancy must be ruled out.

## Background

Oesophageal submucosal tumours originate from mesenchymal tissue and are usually benign. These tumours, which include leiomyoma, stromal tumour, fibroma, and lipoma, are typically covered by normal mucosal epithelium and are usually discovered during oesophago-gastroduodenoscopy (OGD) for other reasons. Oesophageal cancer presenting as a submucosal tumour is very rare. We report a case of oesophageal squamous cell carcinoma mimicking a submucosal tumour.

## Case presentation

**Fig. 1:**
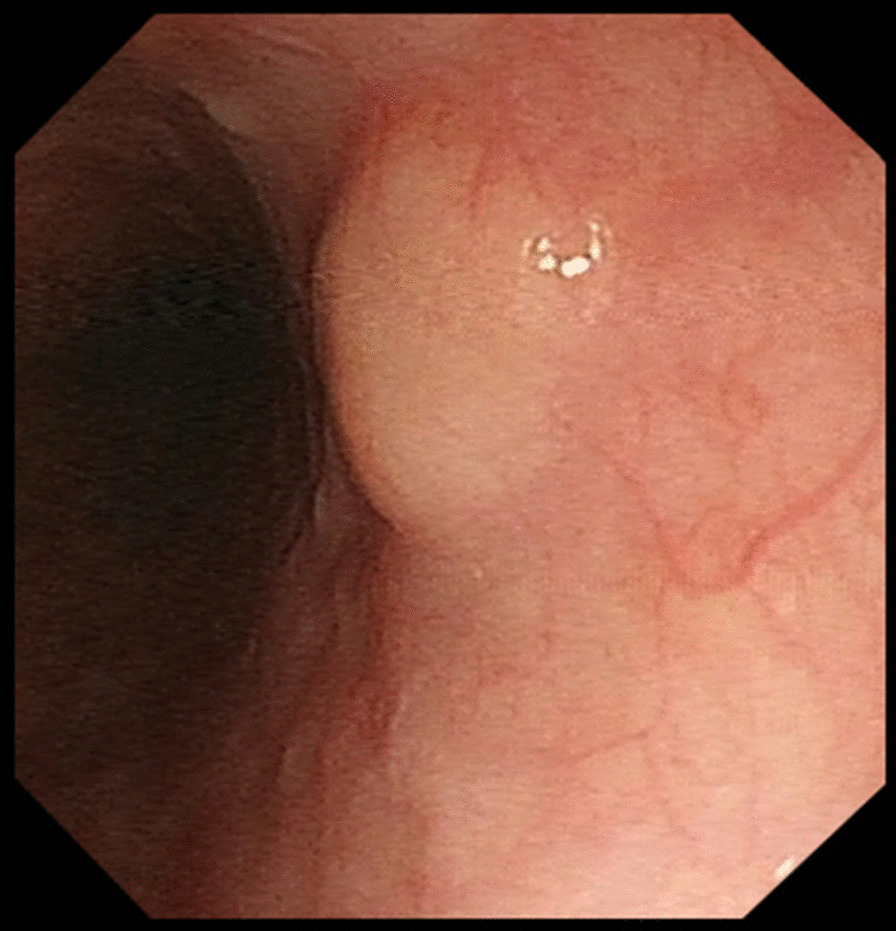
OGD found a smooth-surfaced 0.6-cm sized submucosal tumour in the oesophagus

**Fig. 2:**
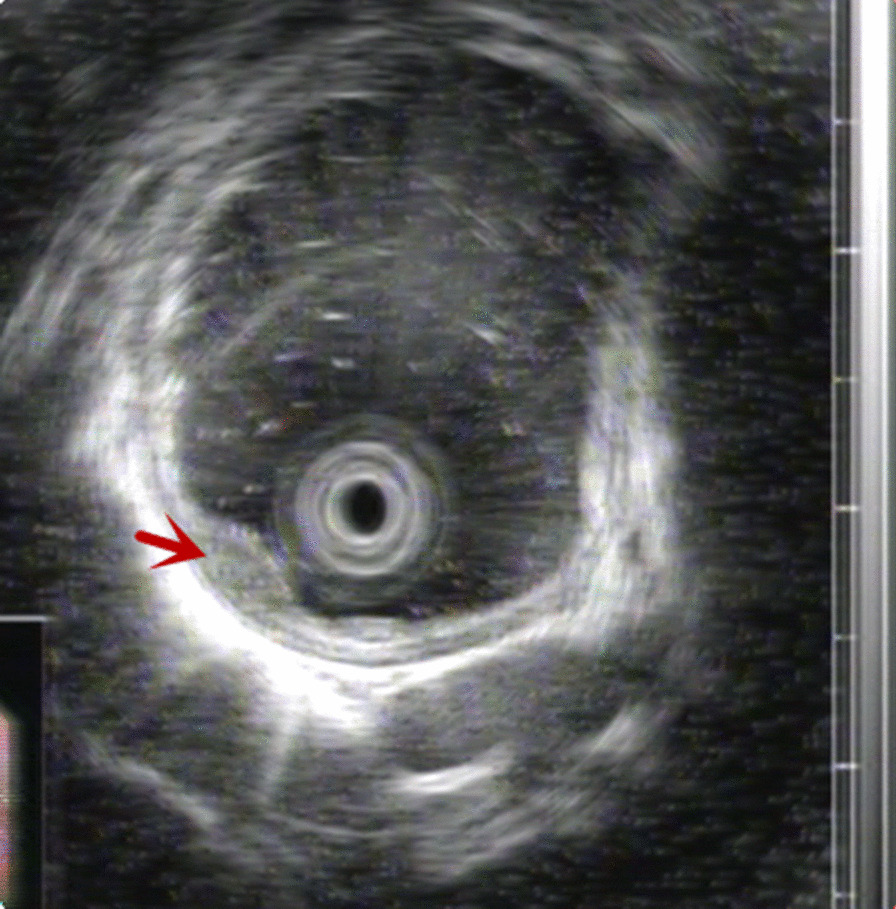
EUS showed a mass arising from the muscularis mucosa

**Fig. 3:**
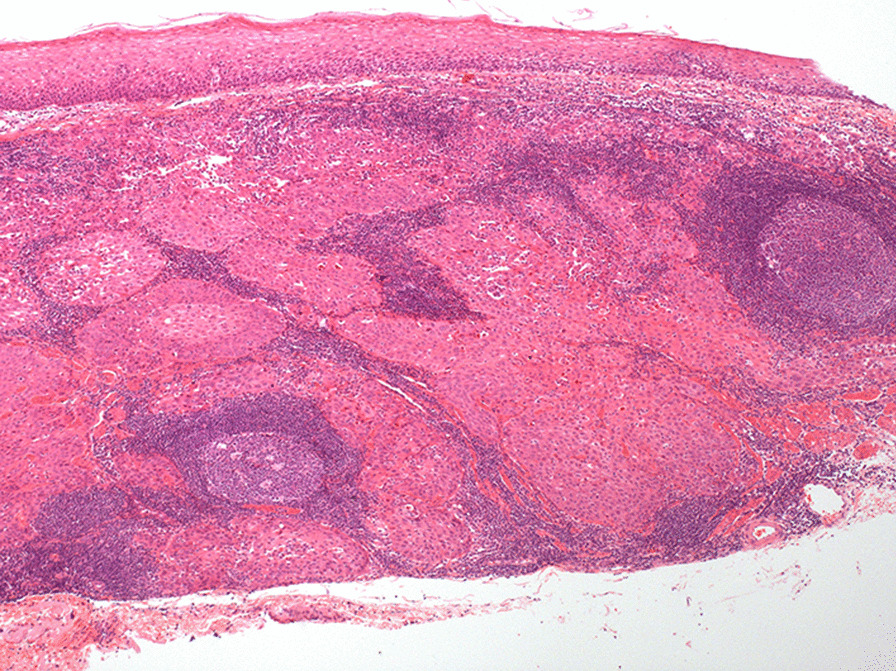
Pathological examination showed it to be a moderately differentiated infiltrating squamous cell carcinoma, with normal overlying squamous epithelium (×100)

A 58-year-old man whose parents had both died of oesophageal cancer underwent screening OGD and was found to have a smooth-surfaced 0.6-cm sized submucosal tumour in the oesophagus located 30 cm from the incisor (Fig. 1). There were no associated symptoms, and physical examination was normal. Chest computed tomography (CT) did not reveal any mass in the oesophagus or enlarged lymph nodes. Endoscopic ultrasonography (EUS) showed a 0.57 cm × 0.22 cm heterogeneously hypoechoic mass, with clear boundary, arising from the muscularis mucosa (Fig. 2). With a provisional diagnosis of leiomyoma, endoscopic submucosal dissection (ESD) was performed. Postoperative pathology showed moderately differentiated squamous cell carcinoma, with invasion of the muscularis mucosae and focal infiltration into the submucosa; the pathological stage was SM1. The cancer tissue was close to the proximal of the lesion, and the distal and basal margins were negative. The cancer was located under the basement membrane, in the muscularis mucosae, and the overlying superficial squamous epithelium was completely normal (Fig. 3). Immunohistochemical staining showed positivity for EMA and CEA in the centre of the tumour cell nest, indicating malignant transformation of mucosal glandular duct epithelium. No evidence of tumour spread to other sites was found on whole-body positron emission tomography computer tomography (PET-CT). Surgical resection of the oesophagus was performed. Postoperative pathology confirmed complete excision of the tumour and no metastasis to dissected lymph nodes.

## Discussion and conclusions

Oesophageal squamous cell carcinoma is a primary oesophageal cancer that usually originates in the oesophageal mucosal epithelium. In the early stages, the mucosa may show congestion; clearly delimited area of redness and erosion; and friability, with a tendency to bleed easily. Ulceration of the mucosa and protrusion into the lumen may also occur.

Oesophageal cancer presenting as a submucosal tumour with normal overlying mucosa is very unusual. Our literature search revealed only three previous reports over the past 10 years. In 2012, Liu et al. reported a 44-year-old woman with oesophageal submucosal tumour that was removed by endoscopic mucosal resection. Pathological examination of the resected specimen showed a well-differentiated mucoepidermoid carcinoma covered with normal stratified squamous oesophageal epithelium [1]. In 2015, Chino et al. reported a case of small-cell oesophageal neuroendocrine carcinoma that resembled a submucosal tumour; however, in this case, the protrusion into the oesophagus was very marked (about 5 cm) [2]. In 2016, Sonthalia et al. reported a middle-aged female patient who presented with progressive dysphagia. OGD showed narrowing of the oesophagus 28 cm from the incisors; the surface mucosa was normal. Enhanced CT showed an inhomogeneously enhancing soft tissue lesion, about 3.2 cm × 2.5 cm in size, almost completely occluding the lumen of the distal oesophagus. EUS showed a solid mass with uneven echo in the muscularis propria. EUS-guided fine needle aspiration cytology revealed the mass to be a squamous cell carcinoma [3]. In 2017, Shibata et al. reported a case of submucosal tumor of the lower oesophageal with a size of 0.5 cm. EUS revealed a hypoechoic tumor originating from the third layer of the oesophageal wall. The pathological diagnosis after ESD was oesophageal gland duct adenoma [4].

Our patient, who did not have any symptoms, underwent routine screening OGD because of the family history of oesophageal cancer. The lesion was only 0.6 cm in size and might easily have been missed. Even when such lesions are discovered, it is common to ask the patient to come for repeat endoscopy, and diagnosis can thereby be delayed. Meanwhile, in view of the related risk factors and epidemiological investigation in the high-incidence area of oesophageal squamous cell carcinoma in China, the patients whose first-degree relatives have a history of oesophageal squamous cell carcinoma are at high risk of oesophageal squamous cell carcinoma [5, 6].

To conclude, an oesophageal submucosal tumor with a smooth surface may not always be benign. Clinicians should be alert to the possibility of malignancy, especially in patients with family history of oesophageal cancer.

## Data Availability

The materials supporting the conclusions of this article are included in the article.

## Abbreviations

OGD: Oesophago-gastroduodenoscopy
CT: Computed tomography
EUS: Endoscopic ultrasonography
ESD: Endoscopic submucosal dissection
PET-CT: Positron emission tomography computer tomography
SM1: Submucosal invasion less than 1000 mm in depth
EMA: Epithelial membrane antigen
CEA: Carcinoembryonic antigen
